# Soil nitrogen dynamics during an oilseed rape (*Brassica napus* L.) growing cycle in a humid Mediterranean climate

**DOI:** 10.1038/s41598-019-50347-1

**Published:** 2019-09-25

**Authors:** N. Villar, M. Aranguren, A. Castellón, G. Besga, A. Aizpurua

**Affiliations:** 1 Department of Plant Biology and Ecology, The University of the Basque Country, Barrio Sarriena s/n, 48940 Leioa, Biscay Spain; 2 NEIKER-Basque Institute for Agricultural Research and Development, Berreaga, 1, 48160 Derio, Biscay Spain

**Keywords:** Plant sciences, Element cycles, Element cycles

## Abstract

Nitrogen budgets help explain the supply pattern of N from the soil to the crop. Through budgeting, an improvement of the N fertilization strategy can be achieved. The objective of the present study, which was carried out under humid Mediterranean climate conditions, was to assess the influence of N fertilization, temperature and soil humidity on soil N dynamics during a whole oilseed rape growing cycle. A field experiment was conducted with two treatments: without N (0 N) and with application of 180 kg N ha^−1^(180 N). Mineralization was calculated from N balances made throughout the growing cycle, all while taking into account measured N uptake by oilseed rape and N losses by leaching and N_2_O emissions. Nitrogen net mineralization was negative after fertilization, reaching –6.73 kg N ha^−1,^ day^−1^_,_ but total net mineralization over the year was similar for the 0 N and 180 N treatments (21 and 8 kg N ha^−1^, respectively). Temperatures over 5 °C were sufficient for initiating the mineralization processes. In the summer, when the soil water content was below the wilting point, immobilization took place; however, there is a risk of N leaching if rainfall occurs thereafter, mainly in the 180 N treatment.

## Introduction

The seed of oilseed rape (*Brassica napus* L.) is commonly used to make oil or add flavour^1^. In addition, its cultivation provides advantages within the rotation of cereals, whose yield can improve up to 10% in the following two years after an oilseed rape crop^2^. A study conducted in France confirmed the positive effect of rapeseed as a previous crop on wheat, showing a yield increase of 600 kg ha^−1^ ^3^. It is also an interesting crop because after obtaining oil from the grain, the residue can be used as animal feed^4,5^. Oilseed rape crops have the advantage of accumulating relevant amounts of N in its tissues, mainly in the stem and leaves during autumn and winter^3,5–7^, which is of significance for the nitrogen cycle. Consequently, a reduction of N leaching into underground waters during that period may be achieved^8–10^ compared to winter cereals.

Taking into consideration both the need to reduce N losses from leaching and the ability to provide enough N for the growing crop as well as for the following one, it is of great interest to quantify the N available for the crop coming from the mineralization of the organic soil N during and after the growing cycle of a given crop. N balances on a field scale or in larger areas are often used to estimate the leaching risk^10^. Thus, a balancing procedure for mineral N in the soil, determining all of the parameters significantly involved therein (mostly N leaching and N uptake by the plant), enables the organic N mineralized to be accurately estimated^11,12^. Thus, N balances are a good methodology for the characterization of N net mineralization, and therefore the values obtained can be used as indicators of the possible risks of N management in agricultural systems^10,13^. In addition, they provide an idea of the nutritional state of a crop and help us to better plan N fertilizer applications.

Mineralization is the process by which organic N is converted into inorganic forms available to plants. Several factors affect mineralization rates. One factor affecting mineralization is the C:N ratio of organic matter present in the soil, such as plant residues after harvest. When these plant residues are high in C content (C:N > 25), immobilization processes take place, but when these residues have a high N content (C:N < 25) N mineralization occurs^14^. In this sense, after harvest of oilseed rape some stubble stays in the field, which is incorporated into the soil during the ploughing for the following crop. This could lead to an initial N immobilization^8,15,16^ that could be released during the following spring^15,16^.

Regarding temperature, there is a significant increase of mineralization when the temperature rises from 15 to 35 °C, with an optimum temperature of 25 °C^17^, but according to some authors, temperatures above 40 °C and below 5 °C cause mineralization to be insignificant^18–21^. Optimum soil moisture for N mineralization is at 80–100% field capacity^17,19,20^, and mineralization is reduced above and below this water content. Soil moisture content regulates O_2_ diffusion that affects aerobic microbial activity^18,19^ and enhances mobility and diffusion of soluble substrates to microbes^18^. Linn and Doran^22^ estimated the maximum microbial activity and consequently an increase of mineralization when soil water-filled pore space (WFPS) is near 60% with a linear increase in microbial activity between WFPS values of 30 to 60%. The interaction between soil moisture and temperature also affects the microbial biomass^19,20^ and consequently mineralization. In this sense, some authors have observed some mineralization at matric potentials below the wilting point when the temperature is optimum^19,23,24^.

Most studies on the effect of temperature and moisture on mineralization have been performed in laboratory conditions^19,20,25–27^. However, some works on N balances under field conditions with oilseed rape have been made^9,28^, but the number of measurements have not been intensive enough to exhaustively study the N mineralization pattern during a whole growing cycle^29^. Apart from that, most of the research about oilseed rape comes from France and northern Europe, and little has been performed in humid Mediterranean conditions in calcareous soils^30,31^. Therefore, the objective of the present study, which was carried out under humid Mediterranean climate conditions, was to assess the influence of N fertilization, temperature and soil humidity on the soil N dynamics during a whole oilseed rape growing cycle.

## Results

### Outputs and inputs of the N balance

#### Oilseed rape N absorption

There were no significant differences in biomass at stem elongation or flowering, but yield and straw biomass at harvest were higher in the fertilized treatment (Table 2). Nitrogen content at stem elongation was not significantly different for both treatments, while at flowering and at harvest total N content was significantly higher in the 180 N treatment. Total N plant uptake at the end of the cycle was 215 kg N ha^−1^ for treatment 180 N and 86 kg N ha^−1^ for 0 N (Table 2).

**Table 1:** Soil properties at the experimental site at the Province of Álava in northern Spain.

	Depth (cm)	
	0–30	30–60
Texture^a^	Clay loam	Clay loam
Sand (g 100 g^−1^)	42	41
Silt (g 100 g^−1^)	27	31
Clay (g 100 g^−1^)	31	28
pH^b^	7.7	7.8
Organic matter (g 100 g^−1^)^c^	2.1	1.6
N (%)	0.2	0.1
C/N	7.7	7.6
P (mg kg^−1^)^d^	60.9	39.6
Active lime (g 100 g^−1^)^e^	1.73	3.47
CO_3_^=^ (g 100 g^−1^)^e^	8.1	18.8
Ca (meq. 100 g^−1^)	33.2	32.3
Mg (meq. 100 g^−1^)^e^	0.6	0.5
K (mg kg^−1^)^e^	175.8	144.5
Rock fragments (g 100 g^−1^)	21.15	32.05
Bulk density (kg m^−3^)^f^	1.58	1.76
Bulk density_wrf_ (kg m^−3^)	1.43	1.52
WFPS Fieldcapacity^g^ (%)	71	72
WFPS Wiltingpoint^g^ (%)	43	42

The soil, which has a clay loam texture, was classified as Hypocalcic Calcisol (skeletic) (IUSS 2007).

^a^Texture (Gee y Bauder^73^), ^b^pH (1:2.5 soil:water), ^c^Organic matter (Walkey^74^), ^d^P (Olsen and Dean^75^), ^e^Active lime, CaCO_3_, Ca, Mg, K (NH_4_AcO, MAPA^76^,), ^f^(Blake and Hatge^62^ modified by Wolf ^63^), ^g^(Saxton and Rawls^65^).

**Table 2:** Aboveground biomass, N concentration, N accumulation, and Nitrogen Nutritional Index of the oilseed rape crop at stem elongation, flowering and harvest (straw and grain) for two N treatments: no N addition (0 N) and application of 180 kg N ha^−1^ (180 N).

	Stem elongation				Flowering				Harvest					
	15/01/2007				24/04/2007				18/07/2007					
	Dry Matter	N		NNI	Dry Matter	N		NNI	Dry Matter		N			
Treatment	(kg ha^−1^)	(g 100 g^−1^)	(kg ha^−1^)		(kg ha^−1^)	(g 100 g^−1^)	(kg ha^−1^)		(kg ha^−1^)	(kg ha^−1^)	(g 100 g^−1^)	(g 100 g^−1^)	(kg ha^−1^)	(kg ha^−1^)
									grain	straw	grain	straw	grain	straw
0 N	2052 A	2.3 A	49 A	0.62 A	4565 A	1.9 A	89 B	0.64 B	2232 B	4852B	0.45 B	2.9 B	64 B	22 B
180 N	2822 A	2.4 A	68 A	0.69 A	8409 A	2.2 A	181 A	0.84 A	3914 A	11805 A	0.79 A	3.1 A	122 A	93 A

Values followed by different letters mean differences between column values (P < 0.05).

**Figure 1:**
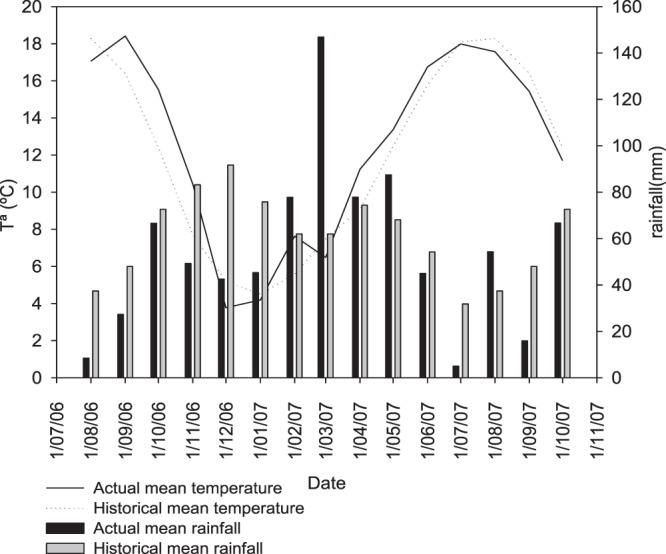
Monthly precipitation (mm) and mean temperature (°C) for the years 2006–2007 and historical data for the period 1970–2006 in the Alava province in northern Spain.

**Figure 2:**
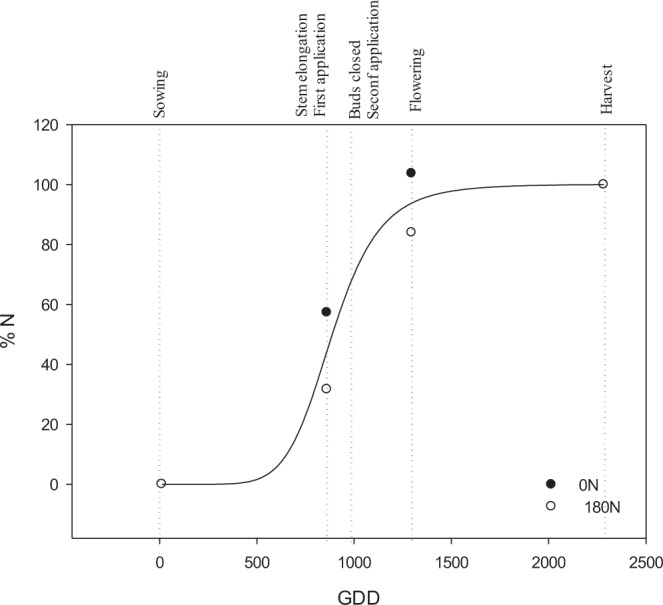
Adjustment of N (%) taken by plants for 0 N (●) and 180 N (○) treatments by a logistic curve to calculate relative N uptake during the whole oilseed rape growing cycle. (GDD, growing degree days).

Nitrogen accumulated by aerial biomass at stem elongation (BBCH31) before the fertilizer application was 49 and 68 kg N ha^−1^, respectively, for unfertilized and fertilized plants, representing up to 57% and 32% of the N uptake by plants (Table 2). From September to December 2006, the mean temperature was 3 °C above the historical mean temperature for this period (Fig. 1), which caused a higher growth of the oilseed rape crop than in other years. From flowering to harvest there was a reduction (3.3%) in the N content at the 0 N treatment and it increased to only 16% at 180 N (Table 2). From flowering onwards (Fig. 2) there was less N uptake by the oilseed rape crop.

#### Soil mineral N

**Figure 3:**
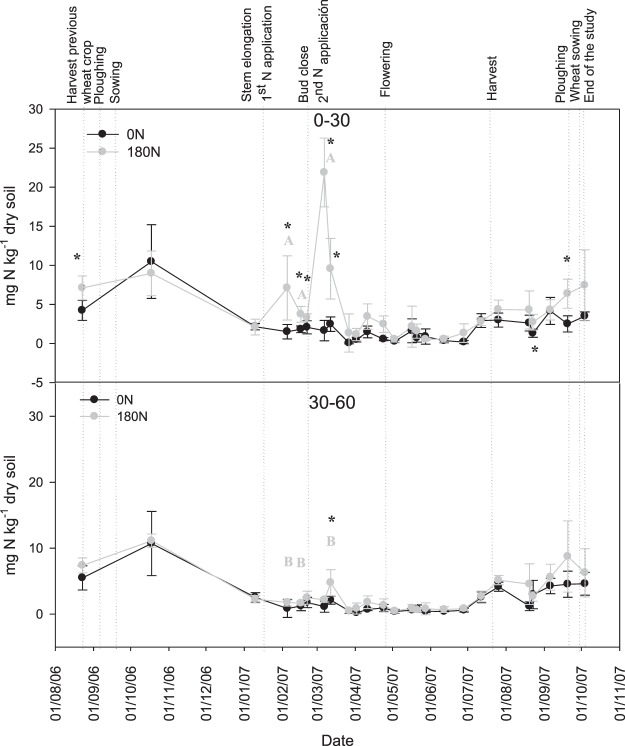
Soil Nmin, at 0–30 and 30–60 cm-layers, from the beginning of August till the beginning of October 2007, comprising the whole rapeseed growing cycle at 0–30 for 0 N and 180 N treatments. Values represent the mean ± standard deviation. Asterisk (*) indicates periods where statistically significant differences between fertilization treatments were found (p < 0.05). Different letters indicate significant differences between Nmin values at 0–30 cm and 30–60 cm depths for N treatment.

There was an increase of mineral N from the harvest of the previous wheat crop to the following sampling data after rapeseed sowing, reaching values approximately 10 mg kg^−1^ for both treatments (Fig. 3). Afterwards, the soil mineral N content decreased to values below 5 mg N kg^−1^ and remained low during the whole cycle, with the exception of the periods after N fertilization. Only from the first of July 2007 did mineral N started to increase progressively, maybe because of the reduction of N absorption by oilseed rape after flowering (Fig. 3). There were no significant differences in soil mineral N between the 0–30 and 30–60 cm layers in the control treatment (Fig. 3), but soil mineral N was higher at 0–30 cm depth in the 180 N treatment immediately after N applications.

Fertilized plots showed higher soil Nmin than control plots at the 0–30 cm depth immediately after N applications, after harvest of the previous wheat crop, and just before the moment of ploughing for the following wheat crop (Fig. 3). In the 30–60 cm layer there were no significant differences in Nmin between treatments, except for immediately following the second application, when a slight increase of soil Nmin was registered in the 180 N treatment; this observation indicates that fertilization hardly affected the 30–60 layer. This fact indicates that the N applied was efficiently absorbed by the rapeseed root system, preventing the expected soil Nmin increase in the 30–60 cm layer.

#### Drainage and leaching

Drainage during the period studied was 461 mm and the concentration of NO_3_^−^ in the drainage water was low (data not shown); it was below the legal limit for drinking water, which is 50 mg NO_3_^−^L^−1^, throughout the whole experiment.

When N was applied during the maximum uptake period, nitrogen fertilization did not cause great amounts of N to be leached at the 180 N treatment (6.98 kg N ha^−1^), and there were no significant differences from the 0 N treatment (Table 3). Until harvest, only 1.41 and 1.28 kg N ha^−1^ were leached from treatments 0 N and 180 N, respectively. Similar low soil mineral N contents at the 30–60 cm layer were found for both the 0 N and 180 N treatments during most of the growing season.

**Table 3:** Nmin leachates below the 60 cm soil layer and N_2_O emissions from rapeseed sowing (18/10/2006) to tillage for the next wheat crop (4/10/2007) for 0 N and 180 N.

Stage	Period	Leachates kg N ha^−1^		Emissions N_2_O kg N ha^−1^	
		0 N	180 N	0 N	180 N
	18/10/2006–10/01/2007	0.15	0.11	0.00	0.00
1^st^ application	10/01/2007–05/02/2007	0.14	0.08	0.00	0.01
	05/02/2007–16/02/2007	0.24	0.20	0.01	0.02
	16/02/2007–21/02/2007	0.04	0.02	0.01	0.01
2^nd^ application	21/02/2007–07/03/2007	0.09	0.11	0.02B	1.16 A
	07/03/2007–12/03/2007	0.03	0.03	0.01	0.15
	12/03/2007–27/03/2007	0.23	0.09	0.01	0.11
	27/03/2007–02/04/2007	0.16	0.05	0.01	0.01
	02/04/2007–11/04/2007	0.09	0.16	0.01	0.03
	11/04/2007–24/04/2007	0.02	0.02	0.01	0.01
Flowering	24/04/2007–03/05/2007	0.04	0.04	0.01	0.01
	03/05/2007–17/05/2007	0.07	0.09	0.01	0.00
	17/05/2007–21/05/2007	0.00	0.00	0.00	0.01
	21/05/2007–28/05/2007	0.00	0.09	0.00	0.00
	28/05/2007–12/06/2007	0.02	0.00	0.00	0.01
	12/06/2007–28/06/2007	0.09	0.14	0.00	0.00
	28/06/2007–12/07/2007	0.00	0.07	0.01	0.01
Harvest	12/07/2007–06/08/2007	0.00	0.00	0.02	0.03
	06/08/2007–20/08/2007	0.00	0.11	0.00	0.00
	20/08/2007–23/08/2007	0.00	0.00	0.00	0.00
	23/08/2007–06/09/2007	2.39	4.93	0.00	0.01
	06/09/2007–20/09/2007	0.01	0.00	0.00	0.01
	20/09/2007–04/10/2007	0.00	0.66	0.00	0.01
	Total	3.81	6.98	0.12	1.58

Values followed by different letters mean differences between column values (P < 0.05).

After the rapeseed harvest, at the end of August 2007 there was an increment in the N leached (Table 3) after heavy rains in that month, which seemed to be higher in the plots that received fertilizer, although they were not significantly different. Despite this high increase, the legal limit of 50 mg NO_3_^−^L^−1^ for drinking water was not reached.

#### Emissions

**Figure 4:**
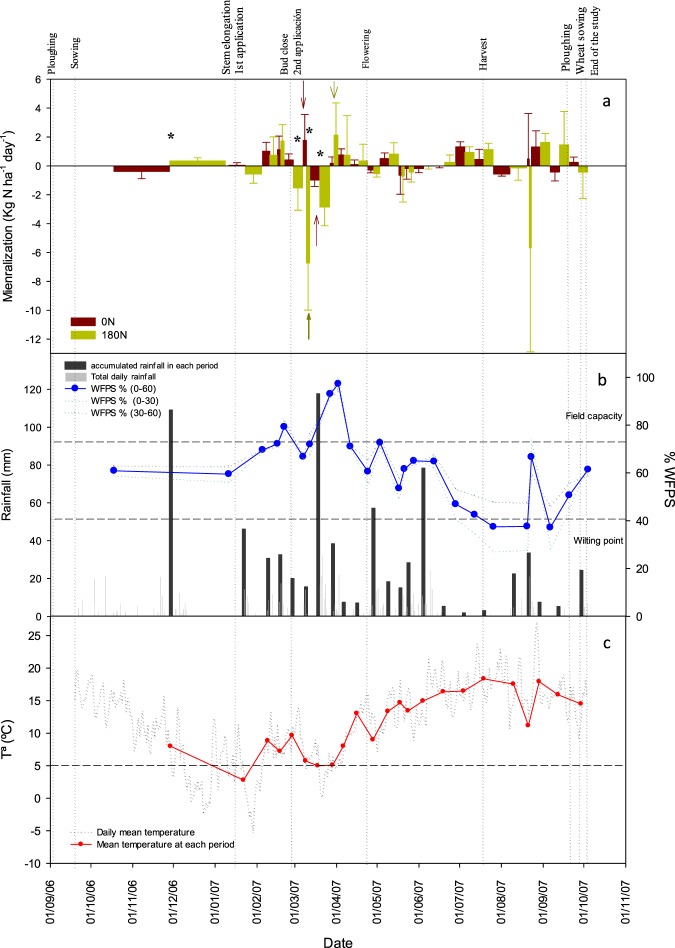
(**a**) Dynamics of soil nitrogen net mineralization (kg N ha^−1^) for 0 N and 180 N treatments from 1/10/2006 to 1/10/2007 throughout the rapeseed growing cycle at a 0–60 cm depth. Mean values ± standard deviation are shown. Asterisk (*) indicates periods where statistically significant differences between fertilization treatments were found (p < 0.05). Arrows indicate the periods of maximum or minimum Nmin in each N treatment (p < 0.05). (**b**) Percentage of water filled pore space (WFPS, %) at 0–60 cm depth for each sampling day, daily rainfall and accumulated rainfall in each period (L m^−2^). (**c**) Mean daily temperature and average temperature for each period (°C).

There were low emissions of N_2_O during the oilseed rape cycle (Table 3), with a total of 0.12 and 1.58 kg N ha^−1^ for the 0 N and 180 N treatments, respectively. Low N_2_O emissions were found following the first N application, probably due to the low temperatures registered (below 0 °C). However, cumulative N_2_O emissions after the first N application at the 180 N treatment were twice as high than in the 0 N treatment. The second N application induced significantly higher N_2_O emissions, with soil temperatures approximately 6 °C and WFPS approximately 80% (Fig. 4).

### Nitrogen budget. Net N mineralization

Mineralization rate for the 0 N treatment varied from −0.97 to 1.77 kg N ha^−1^ day^−1^ and for the 180 N treatment from −6.73 to 2.13 kg N ha^−1^ day^−1^ (Fig. 4).Total net N mineralised was 21 and 8 kg N ha^−1^ for the 0 N and 180 N treatments, respectively, with no significant differences between treatments (P > 0.05).

From mid-October to stem elongation (just before the first N application) the net N mineralization was positive at 180 N treatment but negative at 0 N (Fig. 4). During this period there was a wide range of daily temperatures (from 18 °C to −2 °C).

During the period from the first to the second N application there was a period of time (10 January 2007 to 05 February 2007) characterized by temperatures below 5 °C (Fig. 4). Under these conditions net mineralization in the non-fertilized treatment was null (0.04 kg N ha^−1^ day^−1^), while in the 180 N treatment there was a slightly negative mineralization (−0.57 kg N ha^−1^ day^−1^). After that period of low temperatures, there was an increase until reaching values over 5 °C (after 5 February 2007), when soil moisture was at field capacity. During that time both treatments showed positive net mineralization (Fig. 4).

Just after the second N application, when temperatures were still over 5 °C, the 0 N treatment still showed positive mineralization values until the 12 of March, whereas the 180 N treatment showed a high negative net mineralization rate of −6.73 kg N ha^−1^ day^−1^ (Fig. 4).

In mid-March, both treatments showed negative net mineralization, probably due to a drop in temperatures below 5 °C during those days, and a soil moisture over field capacity, reaching nearly 100% of WFPS (Fig. 4).

During April, just before flowering, with the increase of temperatures, both treatments showed net mineralization (Fig. 4); although not significant, it tended to be higher in the 180 N treatment.

From flowering to harvest, there was a period with negative mineralization values, but when nearing harvest there was a tendency towards an increase in net mineralization in both treatments (Fig. 4), reaching values of 0.45 and 1.13 kg N ha^−1^ day^−1^ for 0 N and 180 N, respectively. During this period, the temperature was above 15 °C, and the soil moisture was below field capacity (48–73% WFPS).

After harvest, net N mineralization was negative at the end of August, reaching values of −5.67 kg N ha^-1^ day^-1^ in the 180 N plots. Although the temperature was optimal (15–25 °C), after harvest the soil remained below the wilting point for almost a month. During this period there was a reduction in the net mineralization. However, during this summer period there was a heavy storm (21 August 2007) that suddenly raised the soil moisture to nearly field capacity. Just after the storm, there was a very negative value of N net mineralization in the 180 N treatment while there was very little mineralization in the 0 N treatment.

Just before ploughing for the next crop, when temperature and moisture were in their optimal range, the 0 N treatment showed net negative mineralization, while there was a quite high net N mineralization at the 180 N treatment (1.45 kg N ha^−1^ day^−1^). After ploughing, both treatments showed values of net N mineralization close to that of 0 kg N ha^−1^ day^−1^.

## Discussion

Increases in rapeseed yield with N fertilization have been observed in many studies, and in the same way, plants that received more N fertilizer had a higher N content at harvest^4,9,32,33^ as our findings confirmed. Oilseed rape growth, and consequently N uptake, during autumn can be quite different between years and regions, because its development depends on the temperature during autumn and winter^34^. In 2016, autumn and winter temperatures were higher than the historical mean temperature for this period (Fig. 1), which caused a higher growth of the oilseed rape crop than in other years. The capacity of oilseed rape to absorb a high amount of N during autumn has also been described in other studies. In the United Kingdom, Barraclough^6^ measured an N uptake in November of 100 kg N ha^−1^ in rapeseed that was sown in August. Sieling *et al*.^9^ in Germany (with a mean air temperature throughout the year of approximately 8.4 °C), in plots where the only source of N was the residual N of a previous experiment with mineral and organic fertilizers, reported that oilseed rape had absorbed 63 kg N ha^−1^ on average at the beginning of spring growth, much more than other crops such as wheat or barley (30 and 24 kg N ha^−1^, respectively). Using ^15^N, Sieling and Beim^35^ also observed that oilseed rape absorbed more N at early stages (70 kg N ha^−1^) compared to other crops such as barley (35 kg ha^−1^) and wheat (18 kg ha^−1^), which was true even when those crops are sown early in autumn^10^. In a sandy soil in Sweden, Engström *et al*.^36^, in several oilseed rape crops sown in mid-August and fertilized with a range of 60–260 kg N ha^−1^, found a mean N uptake of 75 kg N ha^−1^ in late autumn. This high absorption of N in northern countries could be related to earlier sowing dates; thus, the earlier the sowing date, the higher the number of leaves produced and the greater the N uptake by the plant^34^. This early sowing is important in northern countries, where it is important to reach the rosette state before the dormancy period, in order to survive the winter frost.

The lower N uptake from the soil from flowering onwards by oilseed rape was because of the remobilization of N from vegetative to reproductive organs^4,5,7,28,32^. In this respect, oilseed rape is not considered to be an efficient crop, because the remobilization of N is not complete and part of the N remains in the leaves. Those leaves may fall to the soil before harvest, remaining there as residual N susceptible of being mineralized. Taking into account N plant concentration and biomass, the NNI (Nitrogen Nutrition Index) was calculated (Table 2), and according to the critical dilution curve of Colnenne *et al*.^37^, oilseed rape in this study was grown under nitrogen deficit conditions (Table 2) at both studied treatments.

This increase in soil mineral N from the harvest of the previous crop (wheat) to the rapeseed sowing could be related to tillage prior to oilseed rape sowing, which aerates the soil, facilitating the mineralization of the organic matter^38^. In addition, optimal temperature and water content in the soil for soil N mineralization^22^ were registered during that period. This initial increment in Nmin was also described in Sieling *et al*.^9^ in Germany, who saw an increment in Nmin after drilling of oilseed rape. The values of mineral N found in this study after the previous wheat harvest were slightly higher than others found in studies with similar treatments in the same region^39^ but lower than in the neighbouring region of Navarra, where the mineral N ranged from 6 to 30 mg N kg^−1^ ^30^. Soil Nmin values after rapeseed harvest were remarkably lower (approximately 30 kg N ha^−1^) than the ones found in other studies^10^, in which values higher than 100 kg N ha^−1^ were reached.

Nitrogen leaching depends on the concentration of nitrate present in the soil solution and the amount of drainage, which can be very different from one year to another because leaching is affected by climate, soil characteristics and fertilizer rate^40^. Fertilizer application rate seems to have a direct effect on nitrate leaching^40^ and its environmental risks. When fertilizer is applied at the moment and rate that matches the N demand by the plant, there is little risk of leaching. In this study, where N was applied at the period of maximum uptake rate, nitrogen fertilization did not cause great amounts of N to be leached, which is similar to the findings of Leviel *et al*.^29^ in northern France. Nitrate content at the bottom of the studied profile, in our case the 30–60 cm layer, is the most important factor determining nitrate leaching^29^. Similar low soil mineral N contents in the 30–60 cm layer were found for 0 N and 180 N treatments during most of the growing season, so minimal N leaching was expected. This low leaching could be caused by the fact that the oilseed rape was growing under N deficiency according to the NNI at stem elongation and flowering, and it was absorbed very efficiently after the N was added and mineralized during the cycle. In any case, the high demand of N by oilseed rape throughout autumn confirms the suitability of the rapeseed crop to prevent N leaching during the drainage season^8–10,31,36^.

After rapeseed harvest there was an increment in the N leached just after heavy rains in that month, which seemed to be higher in the plots that received fertilizer. This increment in nitrate leaching is in agreement with Ortuzar^39^, who, in the same region for winter wheat, observed that 50% of N losses by leaching happened at the end of summer. Rains at the beginning of September coupled with still high temperatures probably induce an increment in mineralization, with the subsequent accumulation of Nmin due to the lack of a crop that could absorb it^40^. Apart from leaching in late summer, there is a great risk of nitrate leaching after oilseed rape harvest during autumn and winter, as described by other authors in the same region^30^, as well as in northern European countries^28,36^. These authors considered that leaching was most likely due to the amount of easily mineralizable crop residues left by rapeseed after harvest. Moreover, as mentioned before, at this time N uptake by a crop like wheat^10^ is very low; only 10% of the total N is taken up before tillering^41^.

The N_2_O emissions in this field experiment have been thoroughly described elsewhere^42^. With the second N application, significantly higher N_2_O emissions were induced just when soil temperatures were approximately 6 °C and WFPS approximately 80%, and therefore, denitrification was probably the main soil process involved in N_2_O emission due to the anaerobic soil conditions^42^. Nitrous oxide emissions registered during the trial were slightly higher (2 to 5 kg N ha^−1^) than other measures conducted in the same region in a wheat crop^39^.

Total net N mineralised in the 0 N and 180 N treatments was similar to the results found by^29^ in northern France, where 39 and 20 kg N ha^−1^, for non-fertilized and fertilized treatments, respectively (135 kg N ha^−1^ in two spring applications), were seen, and the difference was not significant. In the overall global balance for oilseed rape, it is stated that some phenomenon makes the balance lose precision. First, the oilseed rape plant loses considerable N amounts before maturity via leaf shedding; thus, leaf N losses of up to 45 kg ha^−1^ or approximately 15% of total plant uptake have been reported^5,7^. Thus, the N of dead leaves that has not been incorporated into the soil Nmin during the period of the assay will not be included inside the balance, so the mineralization will be underestimated. Cumulative net mineralization is calculated by the difference, so it carries the errors associated with all other measurements in the N balance^29^. Another important factor is the high variability of soil Nmin among blocks, and we guess that probably one of the greatest errors is associated with the soil Nmin values. The high solubility of nitrate and hence its high mobility is one cause for its high spatial variability. In addition, when common practices of sample handling and analysis of soil have been studied^43^, an analytical error of UV-spectrophotometry up to 5.5 kg NO_3_^−^ ha^−1^ was estimated, as well as a total analytical error including transport, storage and preparation of samples that was in the range of 10 to 15 kg N-NO_3_^−^ ha^−1^. However, the soil Nmin utilization in the balance supposes there is a way to know at every moment the exact situation of the plant available N, allowing control of the final mineralization result of the organic soil N.

From mid-October to stem elongation (just before the first N application), it is not clear if there was any effect of soil moisture and temperature because there was a wide range of daily temperatures. The low mineralization during autumn and winter under an oilseed rape crop coincided with values found by Leviel *et al*.^29^ in France. They also registered negative net mineralization during autumn and winter in a control treatment, which they related to the decomposition of the previous barley crop residues. In our case, wheat was the previous crop, as is typical in our region. The straw C:N ratio is approximately 150 under our edaphoclimatic conditions, which supposes a high value, similar to that observed for barley. It is noteworthy that the straw is usually removed from the plot and thus the stubble is the only remaining plant part buried. In some studies, temperature has been established as a factor affecting mineralization^17,19,20^. Wang *et al*.^20^ showed, in a laboratory experiment, that that in temperatures below 5 °C, net mineralization is reduced to less than 1 mg N kg^−1^ day^−1^, with no differences between lower temperatures; the cause of this is the constraint on microbial activity, even under optimum soil moisture conditions (field capacity). This fact explains the null mineralization in the non-fertilized treatment and the slightly negative mineralization in the 180 N treatment during the period from the first to the second N application where there were days characterized by temperatures below 5 °C. Just after the second N application, when temperatures were still over 5 °C, the 180 N treatment showed a high negative net mineralization rate. Most likely, an immobilization process was happening, which could be caused by the effects of the N application. Laboratory^44^ and field experiments^45–49^ reported a rapid immobilization of N during the days following a mineral N application. In this sense, it has been hypothesized that microorganisms are better competitors than plants for the available ammonium pool added^44,47,48,50^. Thereafter, part of this immobilized N is recovered again, as also happens in laboratory experiments^46,51^, and a higher N is recovered when the N added is higher, as found by Zagal and Persson^51^. Dejoux *et al*.^52^ reported that during spring there is a rapid mineralization of the leaves fallen during winter from rapeseed crops, explaining the net mineralization just before flowering in both treatments.

Around harvest there was a tendency towards an increase in net mineralization in both treatments. During this period, environmental conditions were in the range of the optimum required for N mineralization^18–22^. An increase in mineralization was also found by Leviel *et al*.^29^ from May to harvest in an oilseed rape crop. From the date of measurement of N plant uptake at flowering to the one made at harvest some loss of senescent leaves can occur, as has been stated by several authors, leading us to an underestimation of the real N absorption, and thus the soil mineralization capacity. Malagoli *et al*.^7^ accounted for the N loss from stem elongation to harvest to represent 11.6% of the total N taken up by the plant throughout these two stages. Rossato *et al*.^5^ stated that before pod filling, N loss of dead leaves is just 2% of the total N taken up, but during pod filling this loss corresponded to 16% of the total N cycling through the plant. Most of the N lost in dead leaves occurred at the end of flowering and it increases with the higher N application rates, amounted to up to 20 kg N ha^−1^ when 200 kg N ha^−1^ is applied and 12 kg N ha^−1^ with no fertilization^4^. After harvest, although the temperature was optimum, the soil remained below the wilting point for almost a month, explaining the negative net N mineralization. However, during this summer period there was a heavy storm that raised the soil moisture suddenly to nearly field capacity. The main process following a heavy storm, after the soil has dried, is an immediate immobilization, followed by a restart of mineralization. As we showed, Appel^26^ and Mikha *et al*.^27^ observed a rapid immobilization immediately after rewetting the soil associated with an increase in microbial activity or biomass, which assimilated mineral nutrients to meet the microorganisms’ demand^27^. This effect could be more evident in the 180 N treatment because of the higher yield, which left a larger amount of residues and consequently of N at harvest that was previously left in the form of dead leaves (Table 2).

Ploughing implies residue (stubble) mixing with soil, what could lead to an initial N immobilization^8,15,16^, explaining the values close to 0 kg N ha^−1^ after ploughing. The immobilized N could be mineralised later in autumn or during the following spring^15,16^. Gallejones *et al*.^31^ used an oilseed-wheat rotation to show that rapeseed stubble had some effect on the yield of the subsequent wheat crop. The increase in N supply due to mineralization of oilseed rape residues was not taken into consideration when applying N fertilization, causing an excess of N that led to the lodging of the wheat in the 180 N treatment.

## Conclusions

Net N mineralization dynamics in the soil throughout the oilseed rape growing cycle and after harvest till the next crops are influenced by N fertilization and the moisture and temperature conditions of the soil.

Thus, after the application of the **N** fertilizer, negative net mineralization was observed that lasted over a month after the second N treatment (120 kg N ha^−1^) was applied. The 180 N treatment showed N net mineralization of 21 kg N ha^−1^ relative to the control treatment of 8 kg N ha^−1^. However, the differences were not significant probably due to the high variability of the data inherent to the global budget.

However, when partial budgets were made and daily mineralization rates were calculated, some interesting tendencies were observed:

- In the case of the treatment without N application, net N mineralization was maximal between wilting point and field capacity. In the summer, after the soil had been below the wilting point for a long period, a flush of net mineralization occurred due to an event of heavy rains, which improved soil moisture. At the same time, the highest N leaching of the whole period of study occurred. Therefore, to prevent the loss of this N when there is no crop to absorb N, it would be interesting to use agronomic management practices such as growing catch-crops or the incorporation of crop residues with a high C:N ratio.
- Moreover, it was found that in temperatures below 5 °C, no net mineralization was measured. However, under these specific humid Mediterranean conditions, the average temperature was over that value for much of the winter, allowing the mineralization of the previous crop residues or the soil organic matter mineralization. During autumn and winter there is positive net mineralization, which should be taken into account in the case of organic fertilizers that are commonly applied before sowing in this region.

## Materials and Methods

### Field experiment

A field experiment was carried out at the Province of Araba in northern Spain (42°49′ N, 2°30′W). The climate is classified as humid Mediterranean^53^, with an average temperature of 11.5 °C and an average annual rainfall of 779 mm (Fig. 1). The soil, with a clay loam texture, is classified as Hypocalcic Calcisol (skeletic)^54^. Soil properties are described in Table 1.

The experiment was extensively described in a previous article^31^. Oilseed rape cv. Standing was sown on 19 September 2006, with a density of 73 plants m^−2^. At pre-seeding, 22 kg P ha^−1^ and 35 kg K ha^−1^ were applied together as Fertigafsa (0–20–17). At stem elongation, 15 January 2007, 30 kg S ha^−1^ was applied as MgSO_4_. Oilseed rape was harvested on 18 July 2007, and after that time the roots and 40–50 cm high stubbles remained in the field. After harvest, the field was ploughed on 20 September 2007, and wheat was planted on 4 October 2007, just after preparing the seedbed with a rotary plough.

The experiment was conducted throughout the whole oilseed rape cycle, which was in rotation with wheat (wheat-oilseed-wheat). Two N treatments were applied: no N addition (0 N) and application of 180 kg N ha^−1^ (180 N) in the form of ammonium nitrate (AN) (33.5%). The fertilizer was applied in two splits: one of 60 kg N ha^−1^ at stem elongation, or BBCH 31 (BBCH scale)^55^ on 15 January 2007, and another one of 120 kg N ha^−1^ at inflorescence emergence (BBCH 50) on 26 February 2007. Treatments were distributed on a randomized block design with four replicates.

Previous to the oilseed rape crop, the same N doses (0 kg N ha^−1^ for the 0 N treatment and 60 kg N ha^−1^ at the beginning of tillering and 120 kg N ha^−1^ at the beginning of stem elongation for the 180 N treatment) had been applied to the previous wheat crop during the 2005–2006 season.

### N balance

Nitrogen budgets in 24 periods were calculated by the difference between N outputs (oilseed rape N uptake, N leached and N_2_O emissions, final soil mineral N) and N inputs (N in seeds, atmospheric N deposition, fertilizer application, initial soil mineral N), and they are expressed in kg N ha^−1^. The total net mineralization, from oilseed rape sowing until the sowing of the following wheat crop, was calculated by adding up the mineralization at each period. Mineralization rate (kg N ha^−1^ day^−1^) was calculated dividing the N mineralized by the number of days in each period. Nitrogen volatilization was not included in the balances. We assumed that NH_3_ volatilization from AN fertilization would be negligible^56^, since tests made at the same area on wheat crops demonstrated that volatilization losses do not occur by fertilizing with ammonium sulphate nitrate.

#### N outputs

Oilseed rape N uptake: Oilseed rape plants were sampled to measure the N uptake at three times throughout the cycle: at stem elongation (BBCH 31) before fertilization, on 15 January 2007; at flowering (BBCH 60) on 24 April 2007; and at harvest on 18 July 2007. Two rows of 0.5 m length were sampled at each elemental plot. Plants were cut at soil height at harvest. Plant samples were divided into seeds and straw and dry biomass was determined after oven drying at 70 °C over 48 h. Plant material was ground and sieved through a 1 mm mesh, and N content was determined by Kjeldahl^57^. Nitrogen absorption at harvest was calculated as the sum of the products of the N concentration in seed and straw multiplied by their respective biomasses.

To determine plant N absorption in the remaining periods, it has been considered that cumulative nutrient absorption by crops follows a sigmoidal shape curve^58,59^. In the present study, the three times of measurement were taken into account, and considering the N uptake at harvest as 100%, we determined its value through the oilseed rape growing cycle by adjusting the N uptake at each time to a thermal-time curve. Nitrogen uptake was related to the growing degree days (GDD), with a base temperature of 4 °C, and the function had a sigmoid shape (Fig. 2) of the logistic type^7^. As the relative N uptake was similar for both fertilizer treatments, one single curve was used (Fig. 2).

N leached: Nitrogen leached was measured using porous ceramic cups as described by Lord and Shepherd^60^ from 14 December 2006 to 4 October 2007, when rainfall was over 25 mm or, in any case, every fortnight. Two ceramic cups per plot were installed at a 60 cm depth, below the rooting depth. A soil description made after harvest confirmed the rooting depth estimated initially. Nitrate concentration in water was determined by flow injection analysis (FIA). The first sample was discarded because its nitrate content was considered to be the result of soil alteration when installing the ceramic cups.

Drainage in the 0–30 and 30–60 cm layers was determined according to Campbell^61^, using Eqs. 1 and 2.

1 $${\rm{D}}30={\rm{P}}-{{\rm{ET}}}_{{\rm{C}}}\pm {\rm{VR}}30$$

2 $${\rm{D}}60={\rm{D}}30\pm {\rm{VR}}60$$

where D30 and D60 are the drainage (mm) below 30 and 60 cm, respectively; P is the rainfall (mm), ET_C_ is the crop evapotranspiration (mm), and VR30 and VR60 are the variation of soil water reserves (mm) in the 0–30 and 30–60 cm layers, respectively.

Soil moisture in both layers was determined gravimetrically for the treatments 0 N and 180 N to calculate the variation of soil water reserve. From those data, the water-filled pore space (WFPS) was calculated using Eq. 3.

3 $${\rm{WFPS}}={\rm{H}}\ast {{\rm{BD}}}_{{\rm{wrf}}}/(1-(B{D}_{{\rm{wrf}}}/2.65))$$

where H is soil moisture (g 100 g^−1^), 2.65 is particle density, and BD_wrf_ is soil bulk density for the <2 mm fraction (g cm^−3^) and was calculated as follows:

$${{\rm{BD}}}_{{\rm{wrf}}}=(({\rm{BD}}\ast 100)\ast (1-({\rm{RF}}/100)))/(100-(({\rm{BD}}\ast 100)\ast ({\rm{RF}}/100)/2.65))$$

where BD is the bulk density calculated as the average of four replicates at each layer, using the Blake and Hartge^62^ excavation method, but the volume is measured using polyurethane resin by the Wolf ^63^ method; and RF is rock fragment content (g 100 g^−1^).

Crop evapotranspiration was determined according to the FAO Penman-Monteith method^64^. Nitrogen leached below 60 cm of depth at each plot was calculated as the product of the mean nitrate concentration in the soil solution and the volume of drainage in the 30–60 cm layer (D60).

Soil moisture at field capacity and wilting point (Table 1) were estimated using the pedotransfer function^65^ included in the program “hydraulic properties calculator”^66^.

N_2_O Emissions: Nitrous oxide (N_2_O) measurements were carried out as previously described by Merino *et al*.^42^ Nitrous oxide was measured daily during the seven days after each fertilizer application, every two days the following week, and every time that leaching samples were collected. Nitrous oxide was measured using 3 L PVC hermetic chambers. There were three chambers per plot at 30 mm. Duplicated samples were taken in each of the chambers and stored in 10 mL evacuated blood containers (Vacutainers, B-DTM) immediately after covering the chambers with an airtight lid and then again after 40 min. The N_2_O concentration in these gas samples was measured with a gas chromatograph (Shimadzu GC-9A) equipped with an electron capture 63Ni detector and a stainless-steel column packed with 80/100 Poropak-Q, preceded by a Drierite trap (280 mm long) to remove water.

#### N Inputs

Seed N: Some authors, such as Velthof and Oenema^67^, consider that not taking into account the N of the seeds could lead to an overestimation of mineralization. Because of this, N content in seeds was estimated as the product of the sowing dose (3 kg seed ha^−1^) and the seed N concentration (31 g kg^−1^).

N in atmospheric deposition: The atmospheric N deposition was estimated as described by Gallejones *et al*.^31^ using data from the study of Casado *et al*.^68^, who provided estimates of annual dry and wet deposition in an area near the experimental field (9.39 kg N ha^−1^ and 8.71 kg N ha^−1^, respectively). Dry and wet depositions were added up to obtain the annual total deposition, and afterwards the total amount was divided by the total annual rainfall in the year of the current study to estimate the daily deposition and therefore the deposition in each period.

Soil mineral N: First soil samples for analysing mineral N (N-NO_3_ plus N-NH_4_) were taken when the previous wheat crop was harvested (23 August 2006). Thereafter, samples were taken every time N leachates were collected until the following wheat crop was sowed (4 October 2007). Sampling was performed by collecting five subsamples at each elemental plot from 0 to 30 and from 30 to 60 cm and mixing them thoroughly to determine soil mineral N. Soil samples were extracted in a 1:2 solution of 1 M potassium chloride (KCl), and mineral N was determined by spectrophotometry^69,70^.

### Statistical analysis

The GLM test included in SAS 9.1 statistic software^71^, which was used to identify interactions between factors (time × fertilization) for each studied variable (soil mineral N, N leachates, N emitted, and N net mineralization). When interactions between the two factors occurred (P ≤ 0.05), an analysis of variance was carried out to determine differences between fertilization dose for each period, and period per fertilization dose. A DUNCAN procedure was used to separate means at the P ≤ 0.05 level of significance. Three values of N-NH_4_ were detected as outliers and eliminated using a box plot included in the PAST statistic software^72^. This software considers as outliers values three times smaller or larger than the percentile 25 or 75, respectively.

## Data Availability

The datasets generated during and/or analysed during the current study are available from the corresponding author on reasonable request.
